# Oxytocin and Three Kinds of Dangerous Behaviors in a Romantic Relationship: Playing, Suffering, and Stalking

**DOI:** 10.3389/fpsyt.2020.572654

**Published:** 2020-10-21

**Authors:** Jie-Yu Chuang

**Affiliations:** ^1^Department of Psychiatry, Cardinal Tien Hospital, New Taipei City, Taiwan; ^2^School of Medicine, College of Medicine, Fu Jen Catholic University, New Taipei City, Taiwan

**Keywords:** stalking, oxytocin, depression, relationship, rumination

## Abstract

Romantic relationships are an essential element of healthy living. Although difficulties in love are encountered often, it seems that three kinds of behaviors in a romantic relationship are more susceptible to physical or psychiatric disorders: playing (sexually transmitted disease), suffering (major depressive disorder or suicide), and stalking (violence or homicide). Oxytocin plays an important role in pair-bonding. Elevated plasma oxytocin concentrations have been observed in new lovers when compared with singles. It is hypothesized that those who display these dangerous behaviors in a romantic relationship might possess specific oxytocin receptor gene aberrancy and the resultant deviant pair-bonding pattern is likely to recur in successive relationships. It is postulated that a blunted oxytocin surge might be observed in playing, whereas exaggerated oxytocin surge might be observed in suffering and stalking. The distinction between suffering and stalking might stem from the difference in their aggression tendencies. Those who suffer displays aggression toward self, while those who stalk displays aggression toward others. The exaggerated oxytocin concentrations in people who suffer and people who stalk might not be suppressed by the discouraging attitudes of their partners and might be maintained by rumination. Considering the whole-body influence of oxytocin, intranasal oxytocin application or gene therapy should be used exclusively for those who display these dangerous behaviors and not for the general population. Future research is warranted to confirm this hypothesis with analysis of modifiers such as gender.

## Introduction

PsychINFO and Pubmed were searched for peer-reviewed studies published between the first available year and August 31st, 2020 with the search terms: (“oxytocin”) AND (“relationship” OR “pair” OR “bond” OR “love” OR “mate”). Studies were included if they met the following criteria: (1) published in English; (2) published in a peer-reviewed journal (3) relevant to the hypothesis. The author then examined the titles and abstracts of the retrieved studies to decide the appropriateness of the inclusion.

### Love Has a Profound Mental Health Impact

Love is an essential element of life. Romantic relationships have a profound effect on life. Stable intimate bonds are associated with physical and psychological health, whereas relationship failure is linked to physical and emotional distress (1). Relationship discord has indeed been observed to be the most frequent cause of emotional distress. Breakup or divorce is often associated with depression (2). A link between breakup and the first onset of major depressive disorder has been demonstrated (3).

### Oxytocin in Love

Oxytocin is synthesized in the paraventricular nucleus and the supraoptic nucleus of the hypothalamus, which project into the posterior pituitary where oxytocin is released into the periphery and throughout the forebrain (4). Oxytocin has been nicknamed the “love hormone” due to its association with attachment in mammals. The influence of oxytocin on pair-bonding has been studied most extensively in monogamous prairie voles. Depression-like behaviors in voles were prevented by repeated subcutaneous injection of oxytocin during partner separation. It has been proposed that oxytocin-facilitated synaptic plasticity that strengthens neural communication between partner-specific social engrams and the nucleus accumbens is the essence of pair-bonding (4).

In human studies, elevated plasma oxytocin levels have been associated with early stage of love, affectionate communication, intimacy, and light touch in romantic relationships. In 2012, Schneiderman et al. examined plasma oxytocin levels in 163 young adults and reported that oxytocin levels were significantly higher in new lovers than in single adults (1). However, it should be noted that no strong linear relationship between peripheral and central oxytocin concentrations has been established yet (5).

### Variation in the Oxytocin Receptor Gene

There is a paucity of literature regarding the oxytocin receptor gene. However, studies have provided evidence about the crucial involvement of the oxytocin receptor gene in human pair-bonding. One of the studies showed a significant association between pair-bonding pattern and single nucleotide polymorphism (rs7632287) in the oxytocin receptor gene (*OXTR*) (6). Another study demonstrated that individuals with a greater cumulative risk of several *OXTR* variants exhibit less empathy toward their partners (7). The interaction among gender, oxytocin receptor gene, and the pair-bonding pattern warrants further exploration (7).

## Dangerous Behaviors in a Romantic Relationship

Difficulties in relationships are encountered quite often. However, it seems that three kinds of behaviors are especially susceptible to serious consequences of romantic relationship discord: playing, suffering, and stalking. Those who play may not be satisfied with a single relationship and is constantly seeking new partners. Those who suffer and those who stalk often face discouraging attitudes of their partners (they are indifferent, uncaring, or have already asked for breakups). However, people who suffer tend to be depressed (aggression toward self), whereas people who stalk might harass or threaten their partners (aggression toward others).

According to a 2019 research based on data gathered from 554 participants in the German Family Panel study, behavior patterns in subsequent romantic relationships tend to remain stable (8). Consequently, it is likely that susceptible people might repeatedly display these three dangerous behaviors in the majority of their romantic relationships.

### Playing

Playing is postulated to be defined as feeling empty and constantly seeking new partners in the majority (more than 80%) of the past romantic relationships. People who display playing do not have strong feelings toward their partners which is distinct from suffering and stalking. Despite having significant emotional, familial, and social impacts, only a few infidelity studies have been conducted in humans (9). Based on studies in prairie voles and marmosets, it has been postulated that oxytocin activity may facilitate the preservation of fidelity by reducing the expression of sociosexual behavior toward opposite-sex strangers (10). In humans, two studies have shown that intranasal oxytocin administration stimulates men in a monogamous relationship to perceive their partners' faces as more attractive than other female strangers (11) and to keep a much greater distance between themselves and attractive women (12).

Consequently, it is hypothesized that the player might display a relatively blunted oxytocin elevation during a romantic relationship with frequent incidences of infidelity. Despite having a relatively low level of oxytocin, the player might display an exaggerated dopamine system, which makes the mating cues inherently rewarding. Mating and addiction involve similar neural mechanisms (4) and multiple sexual partnerships were reported to be associated with an increased risk of substance disorders in a large cohort study (13). This oxytocin deviance might stem from oxytocin receptor aberrancy.

### Suffering and Stalking

Suffering is postulated to be defined as obsessively thinking about one's partner or ex-partner with at least one of the depressive symptoms listed in the DSM-V major depressive disorder criteria in the majority (more than 80%) of the past romantic relationships. Stalking is postulated to be defined as following, harassing or threatening one's partner or ex-partner in the majority (more than 80%) of the past romantic relationships. People who display both suffering and stalking are classified to the stalking group as stalking is assumed to be a more advanced stage than suffering (i.e., presumably, stalking follow suffering).

In contrast to people who play, those who suffer and those who stalk are hypothesized to display exaggerated oxytocin surge during a discouraging romantic relationship, which is possibly related to their aberrant oxytocin receptor gene. Two interesting questions arise: how do they maintain their passion despite the disappointing attitude of their partners and what constitutes the difference between the sufferer and the stalker?

To date, most of the research exploring the biological basis of attachment has analyzed data on an individual basis and very few studies have adapted the dyad as the unit of analysis (14). However, results from this limited literature indicate that the exaggerated oxytocin levels of people who suffer and people who stalk could be maintained by their mental preoccupations and they may not be suppressed by the discouraging attitude or low plasma oxytocin concentrations of their partners. The correlation between plasma oxytocin levels of romantic partners was found to be insignificant (1) and elevated plasma oxytocin levels were found to be associated with individual mental preoccupations in a romantic relationship (1). Moreover, lower plasma oxytocin levels of the partner are associated with empathy reduction in the participant (14). Thus, it is possible that the exaggerated oxytocin levels of those who suffer and those who stalk might be maintained purely by their mental preoccupations and they might not be suppressed by their partner's discouraging attitude. These findings may be attributed to their reduced empathy, as they have trouble acknowledging their partners' feelings.

Oxytocin exerts its effect in a context-dependent manner, resulting in either prosocial behaviors or aggression. Consequently, depending on the individual characteristics, exaggerated oxytocin levels might lead to inner aggression (suffering) or outer aggression (stalking).

It is postulated that those who suffer are vulnerable to depression. Rumination is similar to mental preoccupation. It enhances the re-experience of the cognitive, affective, motivational, and physiological consequences of the distress. It has been found to function as a moderator between depressive symptoms and oxytocin (15). Therefore, the association between depressive symptoms and oxytocin seems to be maintained by high rumination levels.

Despite of the limited literature, stalking is a serious problem associated with a high frequency of violence (25–40% of the cases) committed by the stalkers toward their victims (16). Moreover, with the advent of the internet and virtual social networks, cyberstalking has become an emerging problem with devastating impacts on the victims (17) as well as on the stalkers. Stalking usually contains three elements: unwanted following or harassment, credible threat, and inducing fear in the victim. Obsessional thinking (similar to mental preoccupation) is the most common cognitive trait of the stalker. Currently, there has been no clinical research exploring the neurobiology of stalking. However, oxytocin aberrancy has been suspected to be related to the insecure attachment bonding pattern in those who stalk (18).

## Treatment

If left untreated, people who display dangerous behaviors in a romantic relationship might suffer from severe health or social issues such as sexually transmitted diseases, depression, and crime. A large study has shown that depressive symptoms vary according to the related life events. Breakups are associated with high levels of sadness, anhedonia, appetite loss, and guilt. Those who have no history of adverse life events frequently report fatigue and appetite gain, but less sadness (19). Consequently, individually tailored treatment instead of standard treatment might be optimal for people who suffer from psychiatric problems. Therefore, it is hypothesized that oxytocin administration to correct the blunted oxytocin surge in those who play and to maintain oxytocin levels in people who suffer and people who stalk or oxytocin receptor gene therapy to adjust the aberrancy in these people who display dangerous behaviors might be suitable treatment options.

To date, preliminary studies have shown both facilitating effects (such as enhancement of positive communication behavior) and aggravating effects (such as decreased trust in patients with borderline personality disorder) of intranasal oxytocin on romantic relationship (2). This individual difference might be explained by oxytocin receptor gene modulation with respect to sensitivity to oxytocin administration (20).

Oxytocin receptors are expressed throughout the gastrointestinal tract and the heart, suggesting possible whole-body impact of intranasal oxytocin (21). Consequently, it is postulated that intranasal oxytocin should be reserved for these people who display dangerous behaviors in a romantic relationship and not for the general population.

## Discussion

In conclusion, it is hypothesized that people who display the aforementioned types of dangerous behaviors might possess specific oxytocin receptor gene aberrancy, resulting in deviant pair-bonding pattern that is likely to recur in successive relationships. Blunting of oxytocin surge might be found in those who play, whereas an exaggerated oxytocin surge might be observed in people who suffer and people who stalk in a romantic relationship. The exaggerated oxytocin response in those who suffer and those who stalk are possibly maintained by rumination. Intranasal oxytocin application or gene therapy could be considered for these people.

To test this hypothesis, 100 physically healthy volunteers who have just ended a romantic relationship might be recruited and divided into four groups, namely control group, playing group, suffering group, or stalking group based on the aforementioned postulated definitions. Plasma oxytocin concentration measurement and general mental health assessment might be performed for every participant. Half of the participants from the playing group, the suffering group, and the stalking group will receive intranasal oxytocin administration in a randomly assigned and double-blind fashion. Although there is no consensus on the intranasal oxytocin regimen, a systematic review indicates no adverse outcomes when delivered in doses of 18–40 IU for short term use (22). Participants might be administered intranasal oxytocin (24 IU, Syntocinon-Spray, Novartis; 3 puffs per nostril, each with 4 IU oxytocin) (12, 23). Six months later, relationship status assessment, plasma oxytocin concentration measurement, and general mental health assessment of every participant might be performed.

Modifiers such as gender might be considered. It is also necessary to consider the menstrual cycle phases of the female participants in the future study since a meta-analysis has indicated a significant increase of plasma oxytocin concentration from the early follicular phase to ovulation and a significant decrease from ovulation to the mid-luteal phase (24). Furthermore, sexual activity of the participants might also needed to be documented as an increase in plasma oxytocin level during sexual arousal and orgasm in both sexes has been reported in previous studies (1). In short, both intrapersonal and interpersonal fluctuations in oxytocin signaling should be considered in the hypothesis-testing study.

Although oxytocin is believed to be the major neurobiological factor in a romantic relationship, other factors (vasopressin, dopamine, serotonin, cortisol, nerve growth factor, and testosterone) have been reported in the literature. Despite the paucity of related studies, some possible interaction between these factors and oxytocin have been indicated: oxytocin might induce release of dopamine and decrease release of cortisol (25). It is possible to categorize the aforementioned dangerous behaviors based on the sensitivity of these other factors, however this hypothesis is built upon oxytocin reactivity since oxytocin is considered to be the dominant factor in a romantic relationship. Last but not least, romantic relationships are definitely not merely a neurobiological process, other factors might possibly have influences on romantic relationships. For example, the hypothesis is not applicable to the situations when financial dispute is the main reason for the stalking or domestic violence is the principle reason for the suffering.

## Data Availability Statement

The original contributions presented in the study are included in the article/supplementary material, further inquiries can be directed to the corresponding author/s.

## Author Contributions

J-YC developed the hypothesis and wrote the manuscript.

## Conflict of Interest

The author declares that the research was conducted in the absence of any commercial or financial relationships that could be construed as a potential conflict of interest.
